# Synthetic Toll‐Like Receptors for Control of Innate Immunity With Far‐Red Light

**DOI:** 10.1002/advs.202520640

**Published:** 2026-07-27

**Authors:** Anna V. Leopold, Vladislav V. Verkhusha

**Affiliations:** ^1^ Department of Anatomy Medicum Faculty of Medicine University of Helsinki Helsinki Finland; ^2^ Department of Genetics and Gruss‐Lipper Biophotonics Center Albert Einstein College of Medicine Bronx New York USA

**Keywords:** bacteriophytochrome, eDrRTK, IRF3/IRF7, MyD88, NF‐kB, optogenetics, TIR domain

## Abstract

Toll‐like receptors (TLRs) are single‐pass transmembrane proteins that initiate innate immune responses through recognition of pathogen‐associated molecular patterns, including lipopolysaccharide, flagellin, and microbial nucleic acids. In mammals, TLRs are expressed in both immune and non‐immune cells, where they activate cytokine expression through the myeloid differentiation primary response 88 (MyD88) signaling pathway and subsequently engage the nuclear factor kappa‐light‐chain‐enhancer of activated B cells (NF‐κB) or interferon regulatory factor 3 / interferon regulatory factor 7 (IRF3/IRF7) pathways. To enable optical control of TLR function, the extracellular domains of several homodimeric TLRs, including TLR3, TLR4, and TLR5, are replaced with the photosensory core module of the bacterial phytochrome DrBphP. The resulting chimeric receptors activate the NF‐κB and IRF3/IRF7 pathways in mammalian cells in a far‐red‐light‐dependent manner. The MyD88 pathway is further reprogrammed to induce caspase activation instead of cytokine production, thereby creating a synthetic system that links TLR stimulation to caspase signaling. This strategy establishes a versatile optogenetic platform for far‐red‐light control of innate immune and cell death pathways.

## Graphical abstract

Synthetic Toll‐like receptors (TLRs) are engineered by replacing their natural sensing regions with a far‐red‐light‐responsive module from a bacterial phytochrome. These synthetic receptors enable light‐controlled regulation of immune signaling in mammalian cells. Far‐red light serves as a remote trigger for synthetic TLRs, engaging MyD88‐NF‐κB and TRIF‐IRF3/IRF7 signaling axes to precisely control inflammatory cytokine and type I interferon responses.

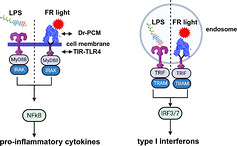

## 1 Introduction

Toll‐like receptors (TLRs) are single‐pass transmembrane receptors that play central roles in innate immunity. Each receptor contains an extracellular domain that recognizes pathogen‐associated molecular patterns (PAMPs), a transmembrane helix, and an intracellular Toll–interleukin‐1 receptor (TIR) domain. Binding of PAMPs, such as lipopolysaccharide (LPS), flagellin, or bacterial and viral nucleic acids, induces TIR‐domain dimerization and initiates downstream signaling, leading to pro‐inflammatory cytokine production through nuclear factor kappa‐light‐chain‐enhancer of activated B cells (NF‐κB) activation via the myeloid differentiation primary response 88 (MyD88) pathway and to activation of interferon regulatory factor 3 (IRF3) and interferon regulatory factor 7 (IRF7) through the TIR‐domain‐containing adaptor molecule / TIR‐domain‐containing adapter‐inducing interferon‐β (TRAM/TRIF) pathway [1].

Cell‐surface homodimeric TLRs, including TLR4 and TLR5, detect bacterial membrane components and primarily trigger NF‐κB–mediated inflammatory responses, whereas intracellular TLRs, including TLR3, TLR7, TLR8, and TLR9, recognize viral and bacterial nucleic acids [1]. Among these, TLR4 signals both at the plasma membrane and in endosomes, activating NF‐κB and IRF3/IRF7‐mediated type I interferon responses, whereas TLR3 primarily activates IRF3/IRF7 signaling [1]. TLRs are expressed not only in immune cells but also in other cell types, including tumor cells, where their effects are highly context‐ and localization‐dependent. For example, TLR4 activation in dendritic cells promotes maturation, lymph‐node migration, and T‐cell activation, supporting antitumor immunity [1]. In contrast, TLR4 activation in tumor cells can promote proliferation, invasion, resistance to apoptosis, induction of immunosuppressive mediators such as nitric oxide and interleukin‐6 (IL‐6) [2], and by excessive stimulation of survival pathways such as mitogen‐activated protein kinase / extracellular signal‐regulated kinase (MAPK/ERK) [3].

Several TLR agonists, including synthetic CpG oligodeoxynucleotides mimicking bacterial DNA, LPS, and synthetic double‐stranded RNA analogs such as polyinosinic‐polycytidylic acid (Poly IC), have been explored as anticancer agents [4]. However, their non‐specific activity can activate TLRs in both immune and tumor cells, potentially limiting therapeutic efficacy. In tumor cells, TLR activation may further promote progression through ERK pathway signaling [2, 5]. These limitations highlight the need for approaches that enable cell‐defined, temporally tunable, and intensity‐controlled TLR activation.

Light‐activatable, genetically encoded receptors offer such control because their expression can be restricted to selected cell types. Far‐red and near‐infrared optical activation further enables high spatial and temporal precision, as shown for receptor tyrosine kinases (RTKs) [6, 7, 8], IsPadC‐derived iLight and iLight2 transcriptional regulators [9, 10], and intracellular protein targeting using RpBphP1‐QPAS1 heterodimerizers [11, 12]. Light‐controlled immune cells capable of cytokine release have also shown therapeutic potential; for example, far‐red light‐controlled immunomodulatory engineered cells (FLICs) secreting interferon‐β (IFNβ), tumor necrosis factor‐α (TNFα), and interleukin‐12 (IL‐12) prevented tumor recurrence in a B16F10 melanoma resection model [13]. However, far‐red optical regulation of immune receptors such as TLRs has not been reported. Existing TLR/NF‐κB optogenetic tools include a blue‐light‐inducible TLR4 based on light, oxygen or voltage (LOV)‐domain‐mediated homodimerization [14, 15] and blue‐light‐induced clustering of cryptochrome 2 (CRY2)‐fused MyD88 and TNF receptor‐associated factor 6 (TRAF6) [16]. These systems either target downstream signaling components or rely on blue light, which has limited tissue penetration, higher phototoxicity, and does not fully recapitulate native receptor‐level signaling.

We previously developed a general strategy for far‐red optical regulation of RTK signaling using the photosensory core module (PCM) of the bacterial phytochrome from *Deinococcus radiodurans* (DrBphP) [17]. In that design, RTK extracellular domains were replaced with DrBphP‐PCM, targeted to the extracellular space by the Igκ secretion signal, and linked to RTK signaling domains through the human epidermal growth factor receptor 2 (HER2) transmembrane domain. This generated far‐red‐light‐activated RTKs, including eDrCKIT, eDrHER2, eDrEGFR, eDrTrkB, and eDrTrkA, that remain inactive in darkness [17]. Because RTKs and TLRs are both single‐pass transmembrane receptors, this receptor‐level strategy provides a logical framework for direct optical control of TLR signaling initiation while preserving membrane topology.

Here, we extend this approach to far‐red light‐inducible TLRs by fusing DrBphP‐PCM to intracellular TIR domains through an artificial transmembrane domain described in [18]. To screen fusions with signaling domains of membrane TLR4 and TLR5 and endosomal TLR3, we optimized an NF‐κB luciferase assay by suppressing negative regulation by suppressor of cytokine signaling 1 (SOCS1) and suppressor of cytokine signaling 3 (SOCS3) using microRNA‐121 (miR‐121) and microRNA‐122 (miR‐222) [19]. This enabled identification of enhanced DrBphP‐PCM‐based eDrTLR4 variants that remain inactive in the dark and are activated by far‐red light, followed by extension of the design to TLR5 and TLR3. We characterized these opto‐TLRs using NF‐κB and IRF3/IRF7 activation assays and western blot analysis. Finally, we used them to optically reprogram the MyD88 pathway by replacing its death domain with the death effector domains of Fas‐associated death domain protein (FADD) (previously called MORT1) or caspase‐8 [20]. Unlike optogenetic strategies that target isolated signaling components, this platform enables light‐dependent activation at the receptor level, preserving pathway branching between the MyD88 and TRIF axes and allowing coordinated control of inflammatory and interferon responses.

## 2 Results

### 2.1 Engineering an Optically ControllableTLR4 Prototype

**FIGURE 1:**
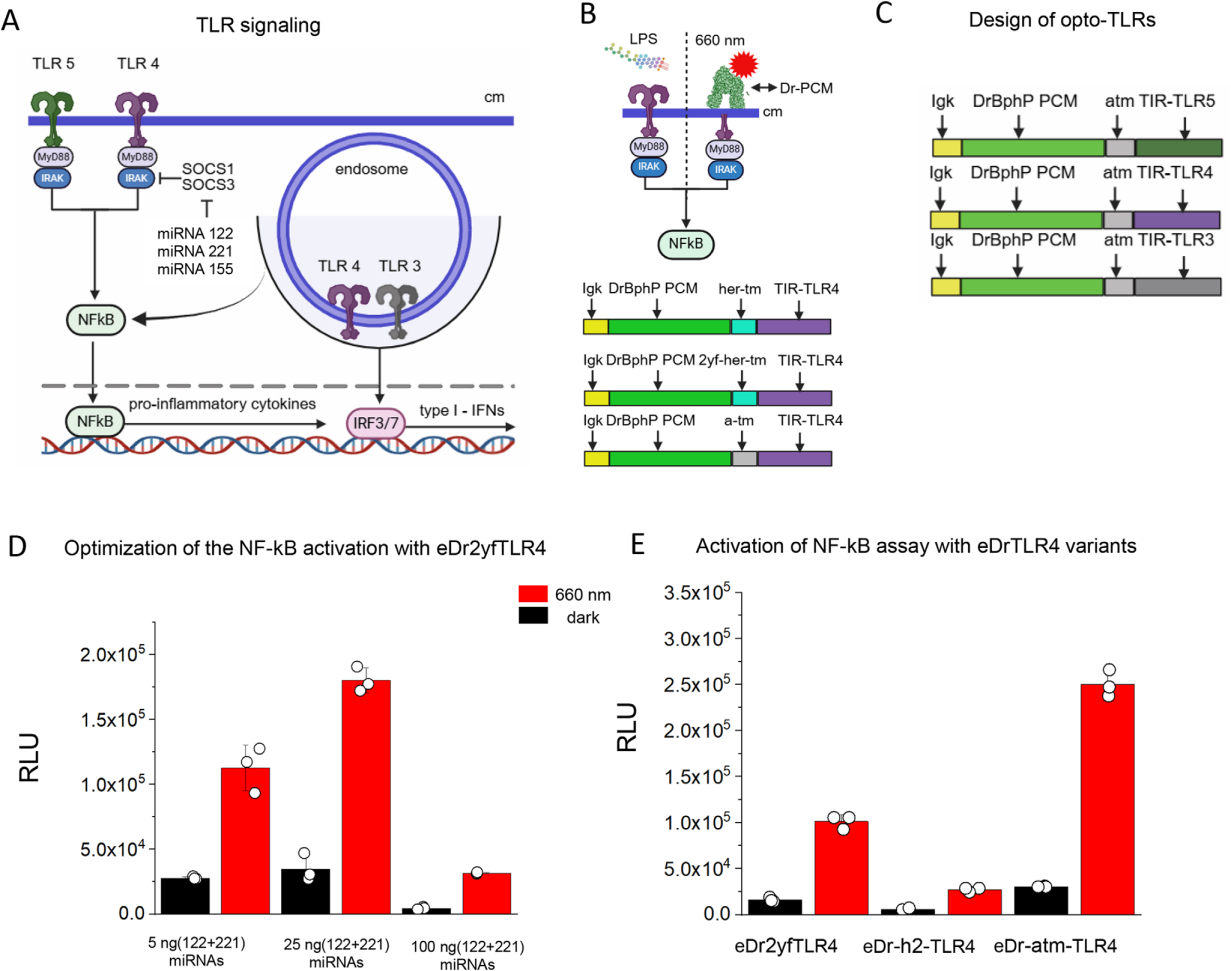
Structural determinants of engineering: comparison of DrBphP and TLRs. (A) Schematic of Toll‐like receptor (TLR) signaling. TLR5 and TLR4 are cell‐surface single‐pass transmembrane receptors, while TLR3 functions as an endosomal receptor. TLR4 can also signal from endosomes. All TLRs primarily activate the NF‐κB pathway. (B) Design of opto‐TLR4. (C) Design of opto‐TLRs. (D) Optimization of NF‐κB activation by eDr2yfTLR4. Increasing microRNA (miRNA) levels suppresses the negative NF‐κB feedback loop. Light‐to‐dark activation ratios are 4, 5, and 7, respectively. (E) NF‐κB activation with eDrTLR4 variants. Light‐to‐dark activation ratios are 6, 4, and 8, respectively. RLU, relative luminescence units. SOCS1 and SOCS3, suppressors of cytokine signaling 1 and 3. miRNA‐121, ‐222, and ‐155, microRNAs that inhibit SOCS expression. LPS, lipopolysaccharide. Dr‐PCM, photosensory core module of DrBphP. Her‐tm, transmembrane domain of HER2 kinase. 2yf‐her‐tm, transmembrane domain of HER2 kinase with the N‐terminal yf repeat. Igκ, secretory signal of Igκ immunoglobulin. Atm, artificial transmembrane domain [18]. TIR, Toll/interleukin‐1 receptor signaling. Diagrams A), B) and C) created in BioRender: A) Leopold, A. (2026) https://BioRender.com/2kefzfk, B) Leopold, A. (2026) https://BioRender.com/e2qeegtA, and C) Leopold, A. (2026) https://BioRender.com/0s7wgi8. Data are presented as mean ± SD from (*n* = 3) independent experiments. No statistical analysis was performed.

TLRs are transmembrane and endosomal pathogen‐associated molecular pattern (PAMP) receptors that can signal as homodimers or heterodimers. Among them, TLR4 is the most well‐studied member of the family [21]. It is a homodimeric, single‐pass transmembrane receptor that recognizes lipopolysaccharide (LPS). Upon activation with LPS, TLR4 induces both NF‐κB and IRF3/IRF7 signaling (Figure 1A). Because TLR4 is homodimeric and well characterized, we selected it as the initial target for developing a light‐controllable TLR prototype.

To achieve this, we followed the workflow previously used for engineering light‐controllable RTKs. We first constructed DrBphP‐PCM fusions containing an N‐terminal immunoglobulin κ chain (Igκ) secretion signal peptide, referred to below as “extracellular DrBphP‐PCM” (eDr), and a C‐terminal transmembrane domain of human HER2 with an N‐terminal YFYF repeat. In our earlier work, far‐red light‐controlled kinase constructs with this YFYF repeat produced the highest efficiency in sustained ERK pathway activation (Figure 1B). Activation of NF‐κB signaling through the TLR4 pathway is regulated by a negative feedback loop that limits signaling after a few minutes (Figure 1A). To maintain a luciferase signal during longer assays, this feedback must be disrupted, as it partially depends on SOCS1 and SOCS3 proteins.

### 2.2 Optimization of the Screening Assay

We then co‐expressed miR‐121 and miR‐222 to inhibit SOCS1 and SOCS3 in PC6‐3 cells. The NF‐κB luciferase assay with varying concentrations of the eDr‐yfyf‐her2‐TLR4 construct demonstrated that the highest light‐to‐dark signal ratio was achieved when maximal amounts of microRNA plasmids were included in the assay (Figure 1D). We also engineered a fusion in which an artificial transmembrane domain, LTVALILGIFLGTFIAFWVVYLL (called “ARTI”), derived from the epidermal growth factor receptor (EGFR) domain [18], served as a linker between DrBphP‐PCM and the signaling TIR domain of TLR4 (Figure 1B). Because eDr‐ARTI‐TLR4 fusions produced stronger activation than eDr‐yfyf‐her2‐TLR4 fusions in the optimized NF‐κB luciferase assay, we selected the artificial dimeric ARTI transmembrane domain for subsequent TLR fusions (Figure 1C).

We next evaluated these fusions for their ability to activate NF‐κB signaling using the optimized luciferase assay. No significant activation of NF‐κB‐dependent luciferase expression was observed in PC6‐3 cells compared with the negative control (Figure 1D). We hypothesized that this was due to negative regulation of TLR signaling by SOCS1 and SOCS3, which can suppress NF‐κB activation through the MyD88 pathway, although they are primarily known as negative regulators of Janus kinase / signal transducer and activator of transcription (JAK/STAT) signaling [22, 23]. To inhibit SOCS1 and SOCS3, we co‐expressed TLRs and NF‐κB–NanoLuc reporters with different combinations of plasmids encoding miR‐121 and miR‐222. The combined use of miR‐121 and miR‐222, in contrast to each microRNA alone or in combination with miR‐155 [24, 25], enhanced NF‐κB‐driven luciferase signaling approximately four‐fold and improved evaluation of the activity of TLR fusions linked to DrBphP‐PCM (Figure 1D,E and Figures S1 and S2).

### 2.3 Expanding the Engineering Approach to Other TLR Variants

After establishing eDrTLR4 and optimizing the NF‐κB transcriptional reporter assay to robustly detect receptor activation, we extended our approach to engineer additional homodimeric TLRs [26], specifically the membrane receptor TLR5 and the endosomal receptor TLR3 (Figure 1C). To generate these constructs, we replaced the cytoplasmic signaling domain of eDrTLR4 with the corresponding intracellular domains of TLR5 and TLR3. TLR5 activates NF‐κB signaling, while TLR3 activates IRF3/7 signaling. Accordingly, only the TLR5 activity was assessed using the NF‐κB assay.

**FIGURE 2:**
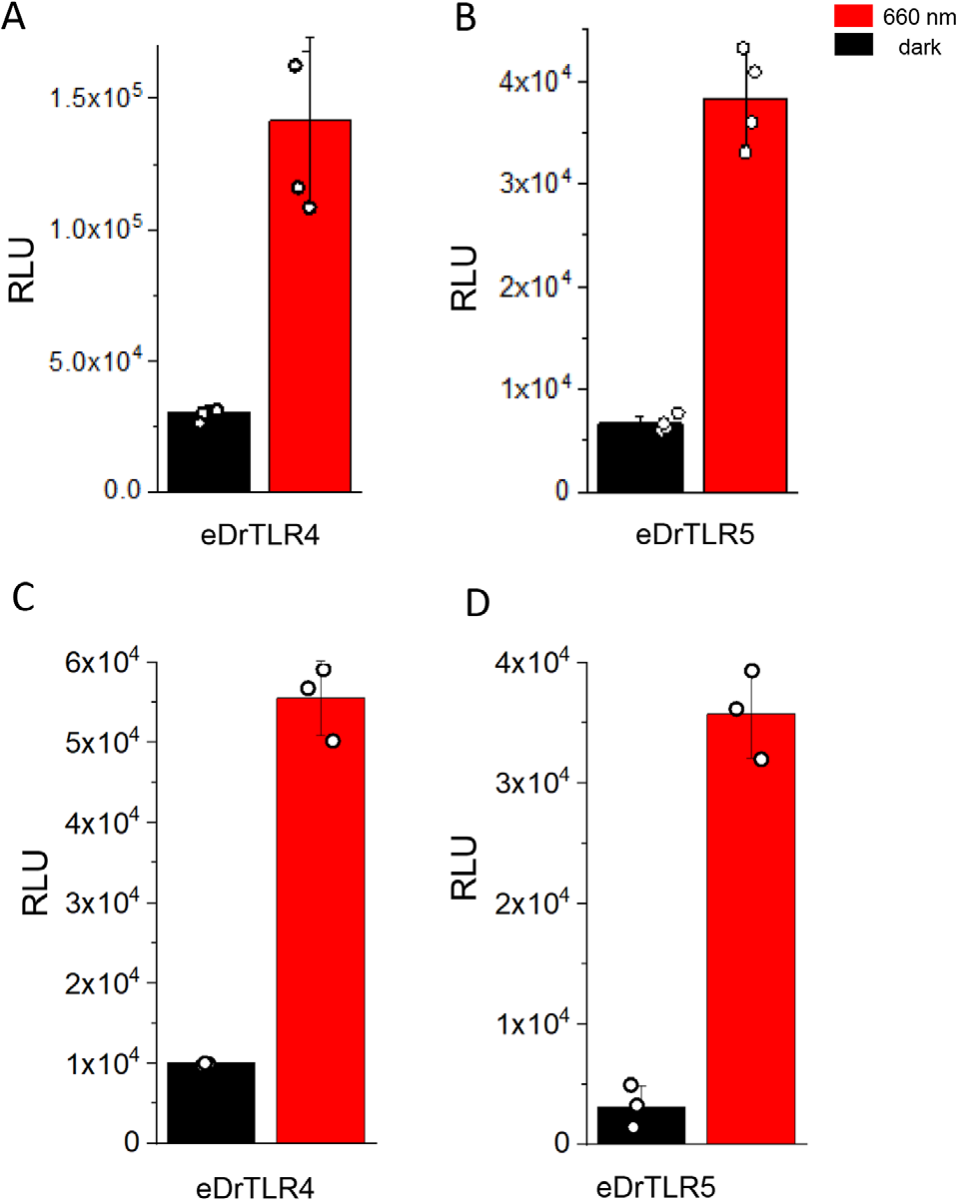
Activation of NF‐κB signaling with eDrTLR constructs. (A–B) NF‐κB‐dependent luciferase activation in PC6‐3 cells. Light‐to‐dark activation ratios are 4.7 and 5.7, respectively. (C–D) NF‐κB‐dependent luciferase activation in HeLa cells. Light‐to‐dark activation ratios are 5.5 and 11.5, respectively. RLU, relative luminescence units. Data are presented as mean ± SD from (*n* = 3) independent experiments. No statistical analysis was performed.

The eDrTLR5 chimeric construct was expressed in the reporter cell system and evaluated with the optimized NF‐κB luciferase assay, which provided a sensitive measure of pathway activation. The TLR5‐based chimera exhibited weaker NF‐κB activation than eDrTLR4 (Figure 2 and Figure S3). This difference can be partially explained by the fact that native TLR4 activates the NF‐κB pathway through both MyD88‐ and TRIF‐dependent signaling. Although the most direct route to NF‐κB proceeds via the MyD88 pathway, TRIF‐dependent signaling provides an additional late‐phase contribution [27], which may account for the stronger response observed with eDrTLR4.

### 2.4 Activation of ISRE Promoter‐Dependent Signaling

**FIGURE 3:**
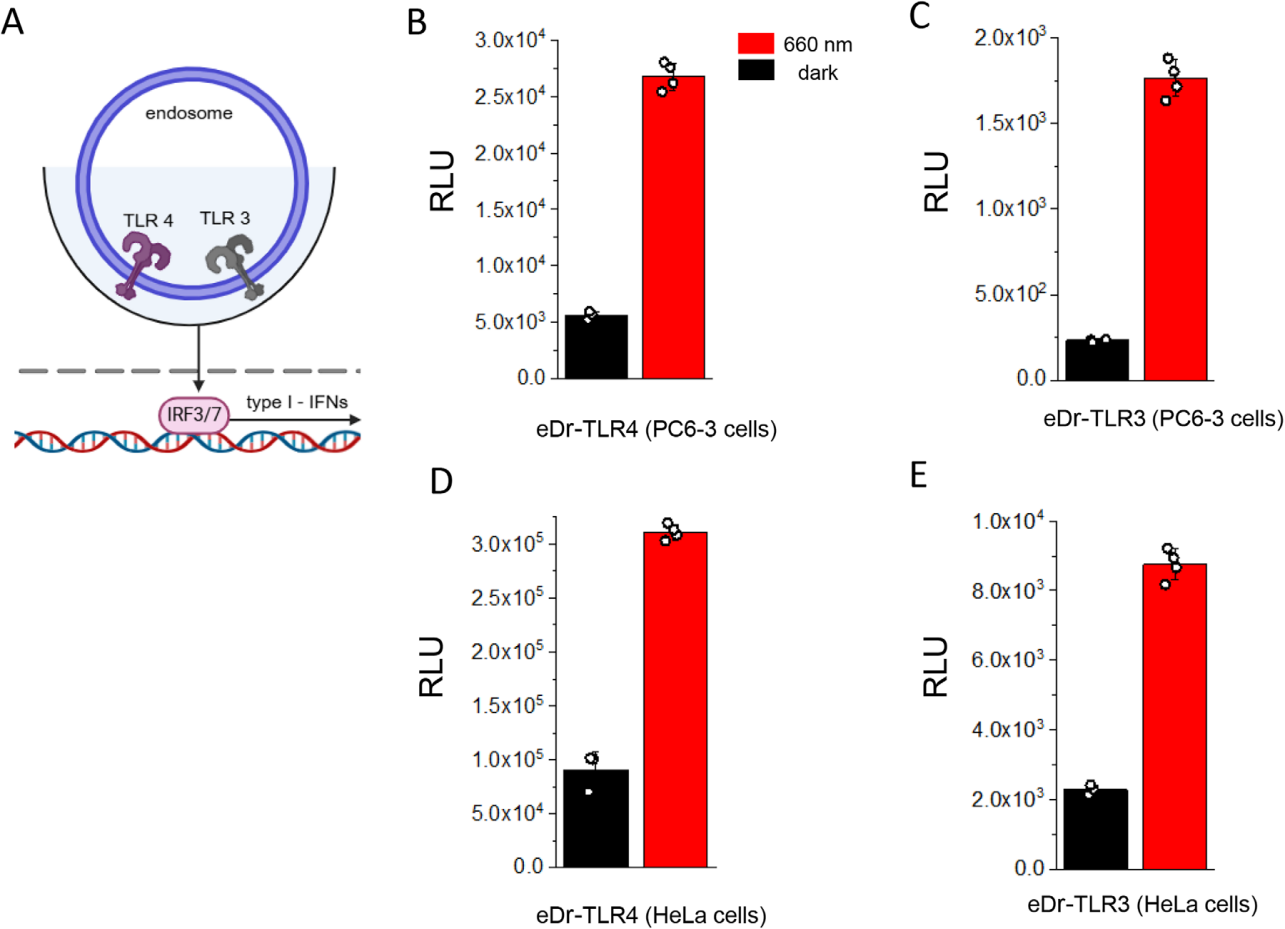
Activation of ISRE signaling with eDrTLR4 and eDrTLR3 constructs. (A) Schematic of endosomal signaling by TLR4 and TLR3. Both receptors activate IRF3/7‐dependent expression of type I interferons (IFNs). (B,C) IRF3/7‐dependent luciferase activation in eDrTLR4‐ and eDrTLR3‐transfected PC6‐3 cells. Light‐to‐dark activation ratios are 4.7 and 7.6, respectively. (D,E) IRF3/7‐dependent luciferase activation in eDrTLR4‐ and eDrTLR3‐transfected HeLa cells. Light‐to‐dark activation ratios are 1.4 and 3.8, respectively. RLU, relative luminescence units. Diagram A) created in BioRender. Leopold, A. (2026) https://BioRender.com/2gaurgh. Data are presented as mean ± SD from (*n* = 3) independent experiments. No statistical analysis was performed.

Because TLR4 can signal from both the plasma membrane and endosomes [1], we tested whether it could activate the interferon‐stimulated response element (ISRE) promoter (Figure 3A and Figure S4), which is regulated by IRF3 and IRF7. For this, we used a plasmid encoding an ISRE‐luciferase reporter to assess IRF‐dependent transcriptional activity.

In parallel, we analyzed TLR3, an endosomal TLR known to signal through the TRIF adaptor pathway, which also activates IRF3 and induces ISRE‐driven gene expression [1]. Both TLR3 and TLR4 constructs were expressed in the reporter cell system and stimulated under conditions promoting TRIF pathway activation.

Our results showed that TLR4 strongly induced ISRE activity, consistent with its role as a TRIF‐pathway activator, whereas TLR3‐mediated ISRE activation was weaker under identical conditions (Figure 3B–E and Figure S4 and Table S1), likely due to the endosomal localization of TLR3. This difference may reflect, on the one hand, distinct expression levels or folding efficiency of eDrTLR4 and eDrTLR3 and, on the other hand, insufficient endosomal localization of eDrTLR3. The N‐terminal signal sequence of eDr targets the relevant constructs to the cell membrane, and whereas native TLR4 can relocalize to endosomes, this process may be less efficient for eDrTLR3. These findings highlight the complexity of endosomal TLR signaling and indicate that precise subcellular localization is essential for efficient IRF3/IRF7 activation and ISRE‐driven transcription. They also suggest that the light/dark ratio might be improved by replacing the secretory localization signal with an endosomal localization signal.

### 2.5 Ability of eDrTLRs to Rapidly Activate NF‐κB Phosphorylation

**FIGURE 4:**
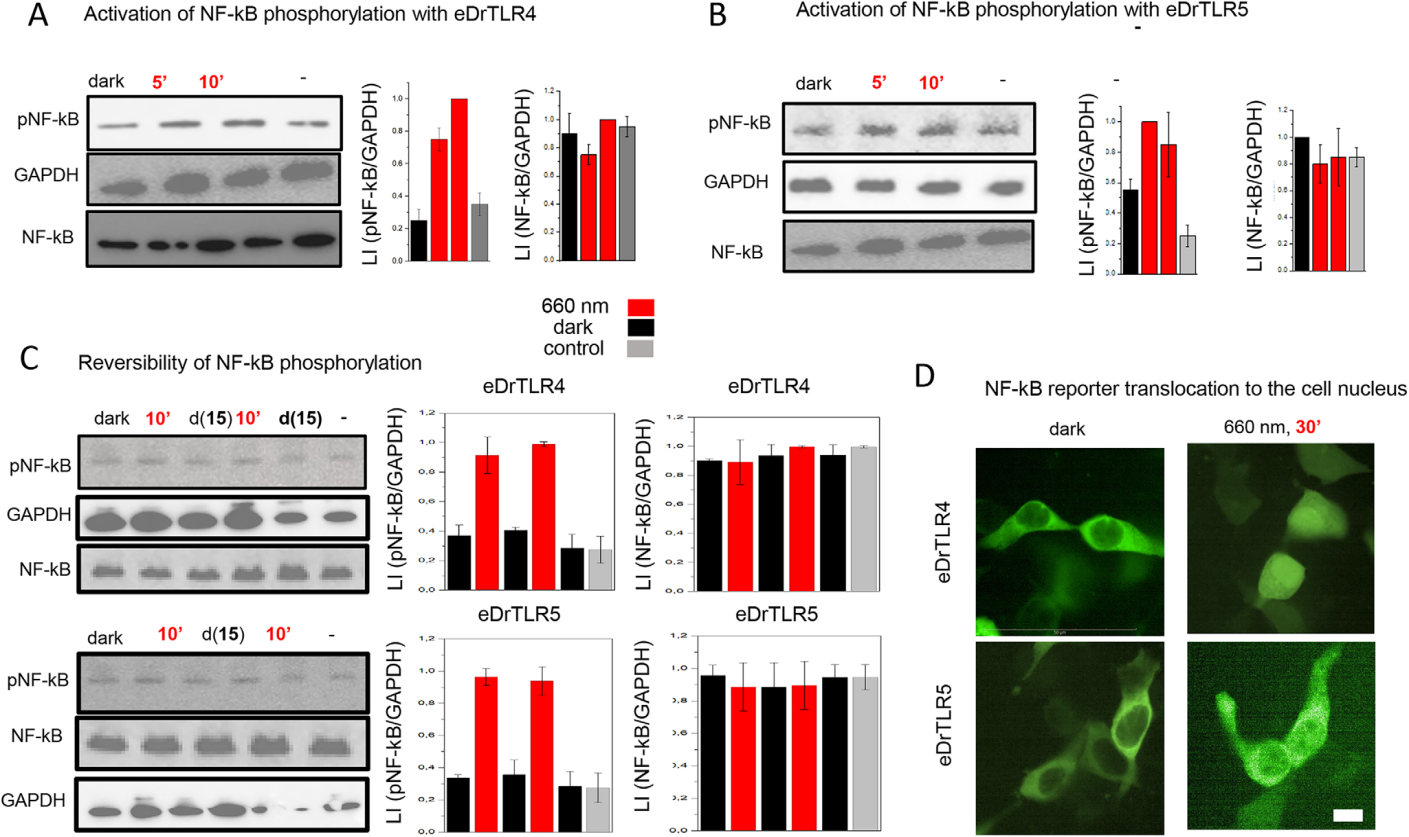
Activation of NF‐κB phosphorylation. (A) Time course of NF‐κB phosphorylation in eDrTLR4‐transfected HEK293 cells under far‐red light. (B) NF‐κB activation after 10 min illumination of eDrTLR5‐transfected HEK293 cells. (C) Reversible NF‐κB activation in HEK293 cells expressing eDrTLR4 or eDrTLR5. (D) Translocation of the NF‐κB fluorescent reporter to the nucleus of HEK293 cells following far‐red illumination. GAPDH, glyceraldehyde‐3‐phosphate dehydrogenase. Uncropped Western blot images are provided in Figures S5 and S6. Data are presented as mean of two independent experiments (*n* = 2). No statistical analysis was performed.

After confirming that engineered enhanced eDrTLRs activate NF‐κB–dependent transcription in luciferase assays, we next validated these results at the protein level by examining NF‐κB phosphorylation, a key indicator of pathway activation. Western blot analysis was used to assess the temporal dynamics of NF‐κB phosphorylation following optogenetic stimulation. HEK293 cells transiently expressing eDrTLR4 were exposed to 660 nm light for 5, 10, or 30 min to determine the optimal activation duration (Figure 4A and Figure S5).

Phosphorylation of NF‐κB was detectable as early as 5 min after illumination, but the strongest and most consistent signal was observed at 10 min. Extending light exposure to 30 min did not further enhance phosphorylation, indicating that maximal activation occurs at 10 min. Therefore, a 10 min illumination period was selected as the standard condition for assessing other eDrTLR constructs, including those based on TLR5 cytoplasmic domains (Figure 4B). This approach enabled direct comparison of phosphorylation profiles among different eDrTLRs and assessment of their relative efficiencies in activating downstream NF‐κB signaling. In addition, TLR4‐mediated NF‐κB activation was shown to be reversible (Figure 4C and Figure S6). Importantly, the observed NF‐κB phosphorylation demonstrates activation of endogenous NF‐κB signaling pathways, thereby providing physiological validation beyond reporter‐based assays.

### 2.6 Translocation of the NF‐κB Reporter to the Cell Nucleus

To confirm that eDrTLR4 and eDrTLR3 trigger nuclear translocation of NF‐κB, we co‐transfected HEK293 cells with eDrTLR4 or eDrTLR5 plasmids along with a fluorescent NF‐κB reporter. Upon 30 min of far‐red illumination at 660 nm, the fluorescent NF‐κB biosensor translocated to the nucleus in HEK293 cells expressing eDrTLRs (Figure 4D).

### 2.7 Reprogramming of the MyD88 Pathway Toward Apoptosis in Cancer Cells

**FIGURE 5:**
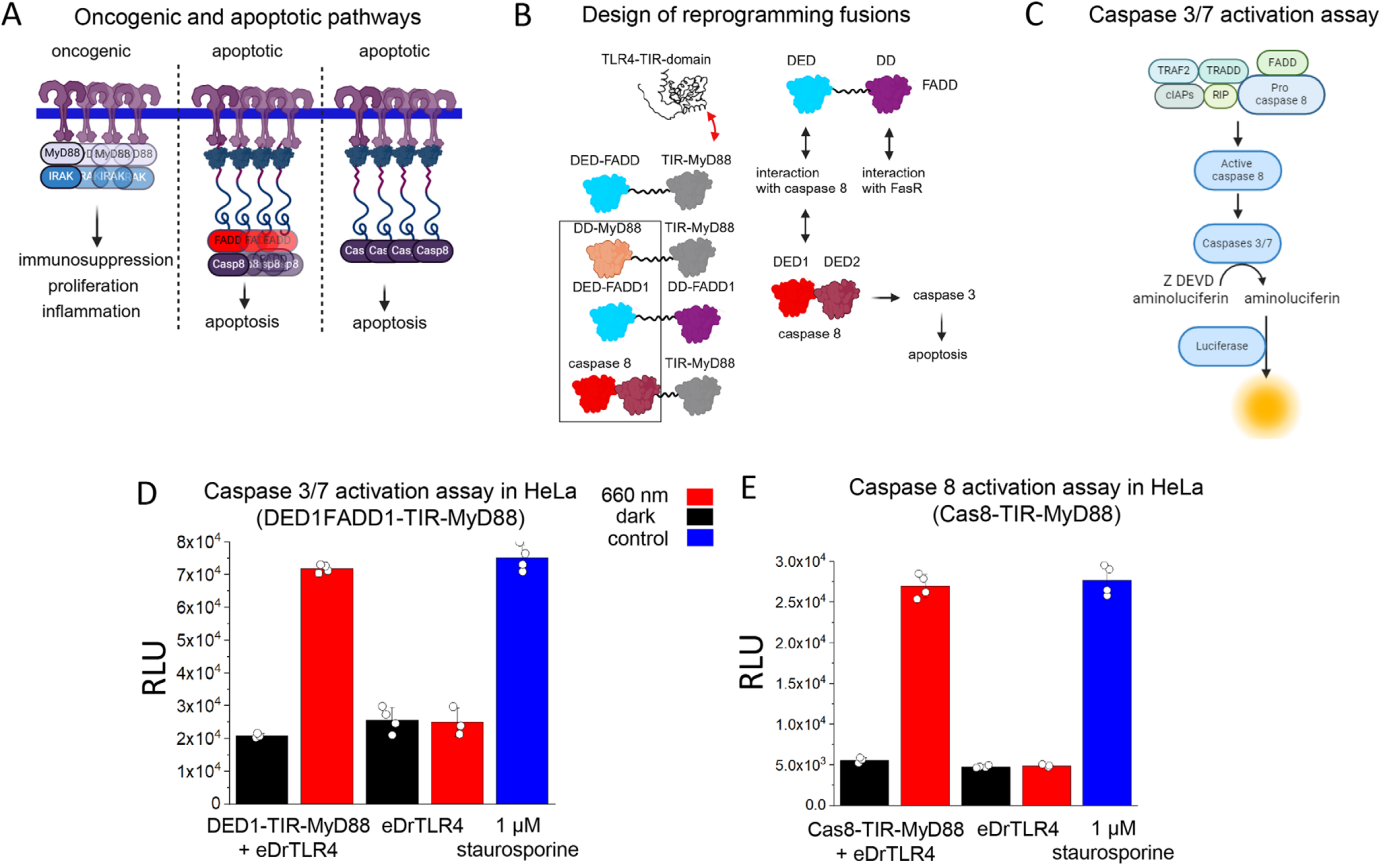
Activation of programmed cell death by MyD88 fusions. (A) Native and reprogrammed TLR4‐mediated signaling. Native oncogenic TLR4 signaling in cancer cells is contrasted with reprogrammed TLR4 signaling. MyD88‐driven polymerization of FADD and Caspase‐8 triggers apoptosis. (B) Design of reprogrammed fusion constructs, in which the death effector domains of FADD or Caspase‐8 are fused to the TIR domain of MyD88. (C) Caspase‐3/7 luciferase activation assay. (D) Caspase‐3/7 activation in HeLa cells co‐expressing DED‐FADD‐TIR‐MyD88 and eDrTLR4. (E) Caspase‐3/7 activation in HeLa cells co‐expressing Caspase‐8‐TIR‐MyD88 and eDrTLR4. RLU, relative luminescence units. DED1, death effector domain 1. FADD1, Fas‐associated death domain protein 1. TIR‐MyD88, Toll/interleukin‐1 receptor domain of MyD88. Z‐DEVD, a caspase‐3/7 synthetic substrate peptide protected at the N terminus by a benzyloxycarbonyl (Z/Cbz) group. Diagrams A), B) and C) created in BioRender: A) and B) Leopold, A. (2026) https://BioRender.com/vzc8vn6. and C) Leopold, A. (2026) https://BioRender.com/y1naxem Data are presented as mean ± SD from (*n* = 3) independent experiments. No statistical analysis was performed.

TLR4 can activate NF‐κB through both MyD88‐dependent and TRIF‐dependent pathways. In the canonical MyD88‐dependent pathway, TLR4 activation induces TIR domain‐mediated homotypic interactions between the receptor and MyD88, which recruits downstream kinases such as interleukin‐1 receptor‐associated kinases (IRAKs), ultimately leading to NF‐κB activation and pro‐inflammatory gene expression (Figure 5A) [15, 21, 27].

To reprogram MyD88‐mediated signaling in PC6‐3 cells, we engineered synthetic fusion constructs linking the TIR domain of MyD88, which preserves oligomerization capacity, to either the death effector domain (DED) of FADD or to full‐length caspase 8 [20]. This design leverages the modular nature of signaling domains: TIR domains mediate receptor–adaptor clustering, and substituting their downstream interaction motifs with apoptotic effector domains redirects signaling from immune activation to programmed cell death.

The FADD DED recruits initiator caspases such as caspase 8 through DED–DED interactions, whereas the direct fusion with caspase 8 enables immediate proximity‐induced dimerization and activation. These constructs were intended to convert TLR4–MyD88 complexes into apoptotic signaling hubs (Figure 5A,B).

We evaluated the pro‐apoptotic potential of these fusions by measuring caspase‐3/7 activity using the Caspase‐Glo 3/7 Assay System (Promega), which provides a sensitive luminescent readout of executioner caspase activation. This assay employs a DEVD–aminoluciferin substrate cleaved by activated caspase 3 and 7, releasing aminoluciferin that is used by luciferase to generate a bioluminescent signal proportional to caspase activity (Figure 5C). The assay follows a simple “add, mix, and measure” format and does not require cell lysis, making it well‐suited for high‐throughput testing. Bioluminescence was quantified with a plate reader. Increased caspase‐3/7 activity in cells expressing MyD88‐TIR–FADD and MyD88‐TIR–caspase 8 confirmed successful redirection of TLR4–MyD88 signaling toward apoptosis (Figure 5D,E).

## 3 Discussion

In this study, we engineered far‐red light–inducible synthetic TLRs, including eDrTLR3, eDrTLR4, and eDrTLR5, by extending our previous DrBphP‐PCM‐based strategy for optogenetic control of RTKs [17] to immune receptors. Specifically, DrBphP‐PCM was fused to intracellular TIR domains through an artificial transmembrane segment (Figure 1A, B), preserving membrane topology, dimerization geometry, and adaptor recruitment. This modular design enabled reversible, light‐dependent, and cell‐specific regulation of TLR signaling, including coordinated activation of MyD88‐ and TRIF‐dependent pathways.

To improve the NF‐κB readout, we used miR‐121 and miR‐222 to suppress SOCS1 and SOCS3, key negative regulators of NF‐κB signaling [19]. This reduced background and increased assay dynamic range. Across constructs, dark‐state activity remained low but detectable, whereas illumination produced approximately 3–5‐fold increases in NF‐κB‐dependent transcription. Stronger SOCS inhibition increased activation contrast up to 7‐fold (Figure 1D and Table S1), indicating that further suppression of negative feedback may improve eDrTLR performance, although lower basal activity will be important for applications requiring tighter off‐states.

Among the engineered receptors, eDrTLR4 produced the strongest NF‐κB activation (Figure 4). eDrTLR3 and eDrTLR5 elicited weaker transcriptional responses, although eDrTLR5 still significantly activated NF‐κB in the luciferase assay (Figure 2 and Figure S3C,D, and Table S1). These differences suggest that linker refinement or addition of an endosomal localization signal to eDrTLR3 may improve performance, particularly because TLR3 signals from endosomal compartments [27].

eDrTLR4 also activated IRF‐dependent signaling, as shown by increased ISRE reporter activity, consistent with its dual role in NF‐κB and type I interferon responses [4, 5]. Variations in basal and induced ISRE activity across constructs and cell types likely reflect differences in endosomal localization and pathway sensitivity. This dual NF‐κB/ISRE activity makes eDrTLR4 a useful tool for probing the balance between inflammatory and interferon‐associated transcriptional programs. Beyond transcriptional outputs, we also reprogrammed MyD88‐dependent signaling by replacing its death domain with FADD or caspase‐8, generating light‐inducible constructs that activated caspase‐3/7 and induced cell death in PC6‐3 cells.

Compared with existing optogenetic tools for NF‐κB control, eDrTLRs provide receptor‐level activation of innate immune signaling, including adaptor recruitment, pathway bifurcation, and feedback regulation. This enables studies of how upstream pattern‐recognition logic shapes downstream outcomes such as cytokine production. However, several limitations remain. The activation magnitude may be sufficient for pathway interrogation but limiting for applications requiring stronger signaling. Dark‐state activity varies across constructs, suggesting incomplete suppression of spontaneous dimerization or signaling. In addition, this study was performed in immortalized cell lines, and performance in primary immune cells or in vivo remains to be established. Finally, although caspase activation is a robust proxy for apoptosis, future studies should include direct viability measurements and additional apoptotic markers, such as annexin V.

In the context of existing optogenetic tools, eDrTLRs complement blue‐light‐based systems by enabling far‐red control at the receptor level, with improved tissue penetration and reduced phototoxicity. This work establishes a platform for spatiotemporal control of innate immune signaling. By enabling selective activation of specific TLR pathways, eDrTLRs can be used to study signaling dynamics, pathway branching, and threshold responses in innate immunity, and may support future immune‐cell engineering strategies after validation in more physiologically relevant systems. eDrTLRs extend the RTK optogenetic platform we previously developed [17] and show that the same design principle, in which a far‐red‐light‐sensitive photoreceptor is fused to the signaling domain of a single‐pass transmembrane receptor, can be applied to immune receptors. More broadly, this approach contributes to translational optogenetics [28, 29], and supports the development of light‐controlled signaling systems for therapeutic [30] and biotechnological [31] applications.

## 4 Materials and Methods

### 4.1 Molecular Cloning

The plasmid encoding full‐length DrBphP was kindly provided by J. Ihalainen. The NF‐κB–NanoLuc reporter plasmid (pNL3.2.NF‐κB‐RE, Cat. #9PIN111) was obtained from Promega. Human TLR signaling domains were amplified from a THP‐1 complementary DNA (cDNA) library. Total RNA was extracted from THP‐1 cells using a silica column‐based RNA purification kit (BioNordika), and cDNA was synthesized with a reverse transcription kit (NewEngland Biolabs).

Mammalian expression vectors were generated by replacing the RTK signaling domains in previously reported eDrHER2 and eDrFGFR1 plasmids [8] using the XbaI restriction site (FastDigest XbaI, Thermo Scientific, Cat# FD0684). An artificial transmembrane domain was inserted by reverse PCR using eDrHER2–TLR4 as a template. During PCR, the XhoI site (FastDigest XhoI, Thermo Scientific, Cat# FD0694) located between DrBphP‐PCM and the HER2 domain was removed. To construct eDrTLR5 and eDrTLR3, the TLR4 domain was replaced with TLR5 and TLR3, respectively, using the same cloning strategy.

The final aminoacid sequences of the engineered eDrTLR3, eDrTLR4 and eDrTLR5 are presented in Supplementary Table S2.

Plasmids encoding miR‐221 (#31519), miR‐122 (#171554), and miR‐155 (#26529) microRNAs were obtained from Addgene.

### 4.2 Cell Culture and Transfection

PC6‐3 cells were cultured in RPMI‐1640 medium supplemented with 10% horse serum (HS; Biowest, Cat. #S0420) and 5% fetal bovine serum (FBS; Biowest, Cat. #S1810). For live‐cell imaging, cells were plated on poly‐L‐lysine–coated coverslips (5 µg/mL; ThermoFisher Scientific, Cat. #A3890401).

For NF‐κB–NanoLuc reporter assays, 20 000 PC6‐3 cells were seeded per well in 24‐well plates with 0.5 mL of growth medium. Cells were transfected with a total of 1 µg DNA (pCMVd2–DrTLR and NF‐κB–NanoLuc reporter plasmid) at a mass ratio of 1:100 using Lipofectamine 2000 (ThermoFisher Scientific, Cat. #11668019) at a reagent‐to‐DNA ratio of 2:1 (µL:µg). For the IRF3/IRF7 reporter assay, PC6‐3 cells were co‐transfected with the same plasmid ratios, substituting the ISRE–luciferase plasmid (pLminP_Luc2P_RE47; Addgene #90385).

Immediately after transfection, cells were illuminated with 660 nm light (0.5 mW/cm^2^) or kept in darkness. The culture medium was not changed during illumination. Cells were incubated for 30 h before further analysis. Following illumination, cells were lysed in buffer containing 50 mm Tris‐HCl (pH 7.5), 10% glycerol, 1% Triton X‐100, and freshly added 1% β‐mercaptoethanol.

### 4.3 NF‐κB Assay

To optimize the NF‐κB reporter assay, various mRNA plasmid concentrations were tested to maximize the light‐to‐dark signal ratio for the eDr2yfTLR4 construct. PC6‐3 cells were seeded in 24‐well plates (20 000 cells per well) 24 h before transfection to achieve 70%–80% confluency. Plasmid DNA was purified using EndoFree Plasmid Purification Maxi kit, Cat. #12362 (Qiagen) to ensure minimal LPS contamination.

Transfections were performed with Lipofectamine 2000 as described above. The total DNA amount per well was fixed at 1 µg, with miRNA plasmid amounts of 12, 30, 60, 120, or 240 ng co‐transfected with eDr2yfTLR4 to maintain constant DNA levels. Cells were then illuminated (660 nm) or kept in darkness for 30 h. NF‐κB‐driven NanoLuc activity was measured as described below. This titration approach identified the optimal concentrations of miRNA plasmids, providing the highest dark/light ratio while maintaining minimal cytotoxicity.

### 4.4 Bioluminescence Assay

After removing the culture medium, cells were frozen at −80°C to facilitate lysis. Cells were then lysed in 100 µL of buffer containing 20 mm Tris‐HCl (pH 8.0), 10% glycerol, 0.1% Triton X‐100, 1 mM PMSF, and 0.1% β‐mercaptoethanol for 30 min at room temperature on a shaker.

NanoLuc activity was measured in 96‐well half‐area white plates (Costar) by mixing 10 µL lysate with 20 µL Nano‐Glo substrate (Promega, Cat. #N1110) and Firefly Luciferase activity was measured in 96‐well half‐area white plates (Costar) by mixing 10 µL lysate with 20 µL Firefly Luciferase substrate (Pierce Firefly Luciferase One‐Step Glow Assay Kit; ThermoFisher Scientific #16197). Bioluminescence was immediately recorded using a Victor X3 multilabel plate reader (PerkinElmer). Data were analyzed with OriginPro 8.6 software.

### 4.5 Western Blotting

HEK293 cells were seeded in 6‐well plates (90% confluence) and transfected with Lipofectamine 2000 in full culture medium containing 25 µM biliverdin IXα (Sigma‐Aldrich). Transfection mixtures were prepared by diluting 4 µg DNA in 400 µL Opti‐MEM (pH 7.4) and mixing with Lipofectamine 2000 at 2 µL per µg DNA. For NF‐κB (p65) phosphorylation assays, eDrTLR4 plasmid was co‐transfected with pEGFP‐N1 at a 1:5 mass ratio.

After 24 h, the medium was replaced with DMEM containing 0.5% fetal bovine serum (FBS) and no biliverdin. Cells were kept in darkness for 4 h and then illuminated with 660 nm light for 5, 10, or 30 min. The 10 min illumination period was used in subsequent experiments. Following stimulation, plates were placed on ice, washed once with ice‐cold phosphate‐buffered saline (PBS), and lysed in 300 µL ice‐cold RIPA buffer (ThermoFisher Scientific, Cat# 89900) supplemented with phosphatase (Cat. #88265) and protease inhibitors (Cat. #A32953). Lysates were clarified by centrifugation at 12,000 rpm for 20 min at 4°C.

Equal lysate volumes (20 µL) were resolved on 10% SDS–PAGE gels, transferred to nitrocellulose membranes, and blocked in 5% non‐fat milk in Tris‐buffered saline (TBS) (pH 7.4) for 2 h at room temperature. Membranes were incubated overnight at 4°C with anti–phospho‐NF‐κB (Ser536) p65 (Cell Signaling Technology, Cat. #3033, 1:2000) and anti–NF‐κB p65 (Cat. #8242, 1:1000). After washing in TBS–Tween (0.05%), membranes were incubated for 2 h with HRP‐conjugated secondary antibodies: goat anti‐rabbit IgG–HRP (Cell Signaling Technology, Cat. #7074, 1:3000) and goat anti‐mouse IgG–HRP (SantaCruz Biotechnology, Cat. #sc‐2005, 1:1000).

Signals were visualized using Clarity Western ECL substrate (Bio‐Rad), and images were acquired with a ChemiDoc Imaging System (Bio‐Rad).

### 4.6 Caspase 3/7 Luciferase Assay

Cells were seeded in 96‐well white, glass‐bottom plates (Costar) at 10,000 cells per well in 100 µL of medium containing 25 µm biliverdin. Cells were co‐transfected with eDrTLR4 and MyD88–TIR–caspase‐8 or FADD–DED fusion plasmids at a 1:3 mass ratio using Lipofectamine 2000. The following day, cells were illuminated with 660 nm light for 6 h to induce apoptosis. Staurosporine‐treated wells served as positive controls.

After treatment, 100 µL of Caspase‐Glo 3/7 reagent (Promega, Cat. #G8090) equilibrated to room temperature was added directly to each well. Plates were shaken for 30 s at 300 rpm and incubated for 30 min at room temperature in the dark. Bioluminescence was measured with a Victor X3 plate reader (PerkinElmer). Background signal from reagent‐only wells was subtracted, and values were normalized to untreated controls. Data were analyzed using OriginPro v.8.6 software.

### 4.7 Statistical Analysis

Data are presented as the mean ± standard deviation (±SD). Each experiment was performed in two or three independent replicates. Mean values and SD were calculated using OriginPro v.8.6 software (OriginLab Corporation). No statistical data transformation or outlier exclusion was performed. Western blot band intensities were quantified by densitometry using ImageJ v.1.54t software (National Institutes of Health, USA). The intensity of each target protein band was normalized to the corresponding glyceraldehyde‐3‐phosphate dehydrogenase (GAPDH) band intensity. For luciferase assays, raw bioluminescence values were measured, and the signal obtained from the negative control was subsequently subtracted from all measurements before data presentation in the main text figures. The corresponding raw bioluminescence plots are provided in the Supporting Information. No formal statistical hypothesis testing was performed; therefore, no *p* values or significance testing are reported.

## Author Contributions

A.V.L. performed the experiments, wrote the manuscript, and drew the figures. V.V.V. conceived the idea, supervised the project, planned the experiments, revised the manuscript and figures.

## Funding

This work was supported by grants GM122567 from the US National Institutes of Health, 220011 from the Jane and Aatos Erkko Foundation, 360277 from the Research Council of Finland, and the Cancer Foundation Finland.

## Conflicts of Interest

The authors declare no conflicts of interest.

## Supporting information




**Supporting File**: advs76818‐sup‐0001‐SuppMat.docx.

## Data Availability

All data supporting the findings of this study are available within the paper and Supplementary Information. The plasmids constructed in this study, along with their full maps and nucleotide sequences, are available at the Addgene repository under the numbers 260460, 260461, and 260462.
