# Supplementary material for: Synthetic Toll‐Like Receptors for Control of Innate Immunity With Far‐Red Light
Source: Adv Sci (Weinh). 2026 Jul 27:e20640. Online ahead of print. doi: 10.1002/advs.202520640 (PMC13403374; doi:10.1002/advs.202520640)
Supplement: Supplementary file 1 — Supporting File: advs76818‐sup‐0001‐SuppMat.docx. [file ADVS-9999-e20640-s001.docx]

**Synthetic Toll-Like Receptors for Control of Innate Immunity with Far-Red Light**

Supplementary Information


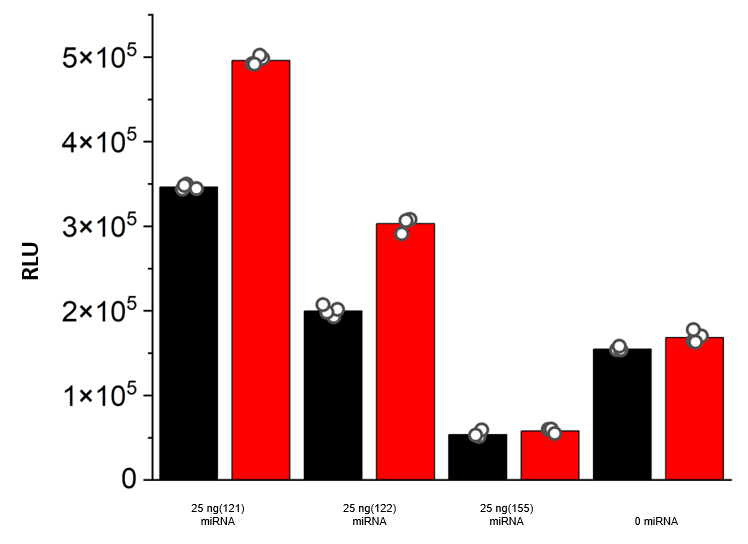


25 ng

(221 miRNA)

25 ng

(122 miRNA)

25 ng

(155 miRNA)

**Supplementary Figure 1.** Activation of NF-kB-controlled luciferase expression with eDr2yfTLR4. The miR-122 and miR-**2**21 miRNAs block the negative SOCS1-connected feedback and enhance NF-kB signal, while miR-155 does not affect. Red – under 660 nm illumination, black – under dark treatment. RLU – relative luminescence units. Data are presented as mean ± SD from (n=3) independent experiments. No statistical analysis was carried out.

**
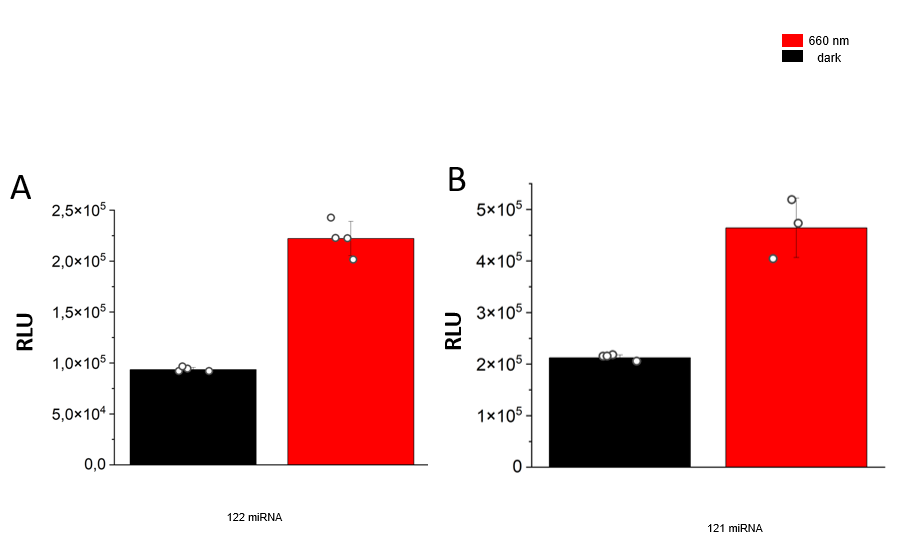
**


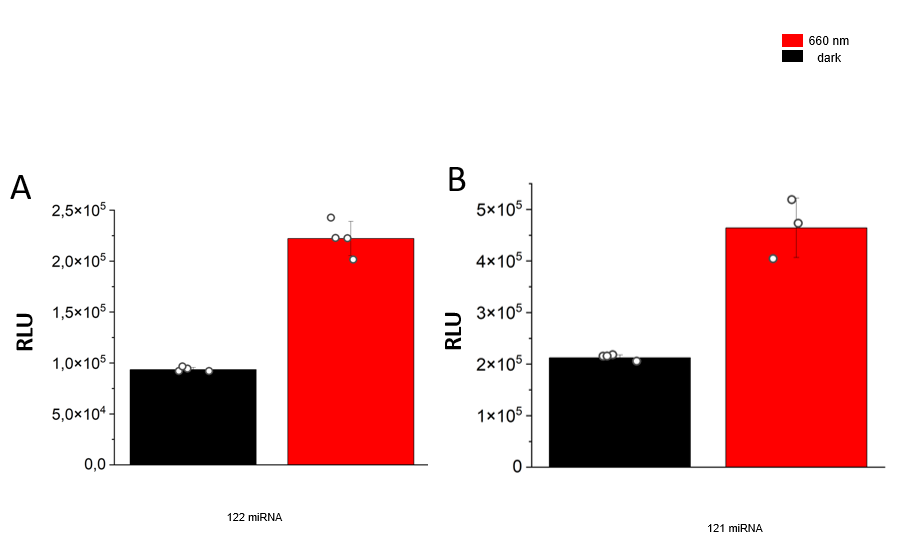


221 miRNA

122 miRNA


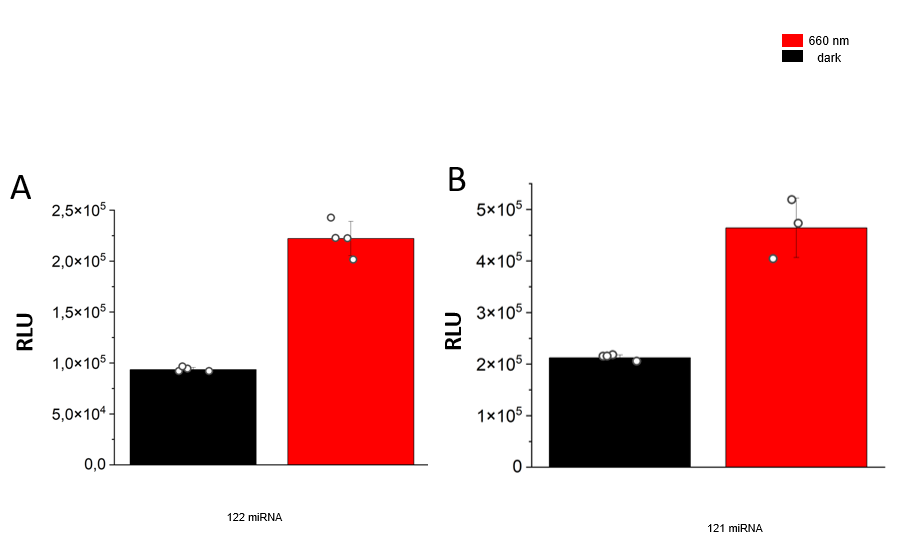


221 miRNA

122 miRNA

**Supplementary Figure 2. The action of individual miR-122 and miR-221 miRNAs on the eDrTLR4 signaling.** eDrTLR4 activates NF-kB signaling upon activation with far-red light. Red – under 660 nm illumination, black – under dark treatment. RLU – relative luminescence units. Data are presented as mean ± SD from (n=3) independent experiments. No statistical analysis was carried out.


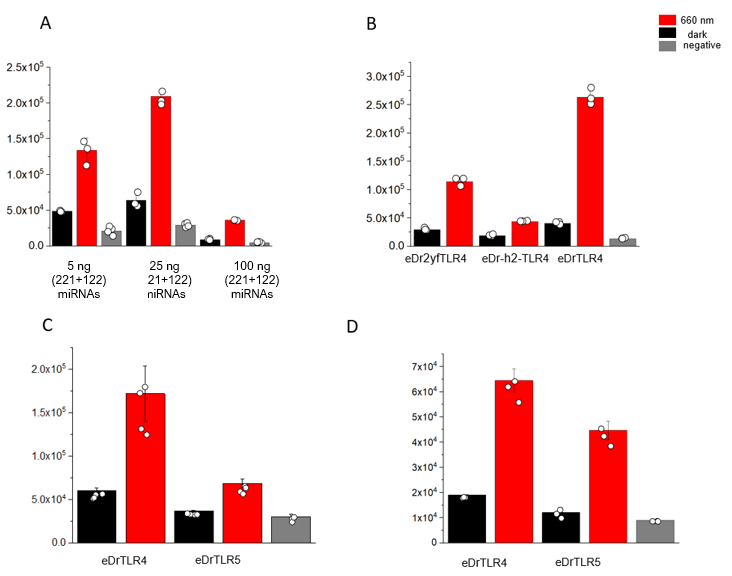


**Supplementary Figure 3.** Activation of the NFκ-B signaling in mammalian cells co-transfected with eDrTLRs and pEGFP-N1. **A)** Optimization of NF-κB activation by eDr2yfTLR4. Increasing miR-122 and miR-221 miRNA levels suppresses the negative NF-κB feedback loop. **B)** NF-κB activation with eDrTLR4 variants. **C)** NF-κB activation with eDrTLR4 and eDrTLR5 in PC6.3 cells. **D)** NF-κB activation with eDrTLR4 and eDrTLR5 in HeLa cells. The Y-axes in all panels represent the relative luminescence units. Data are presented as mean ± SD from (n=3) independent experiments. No statistical analysis was carried out.

**
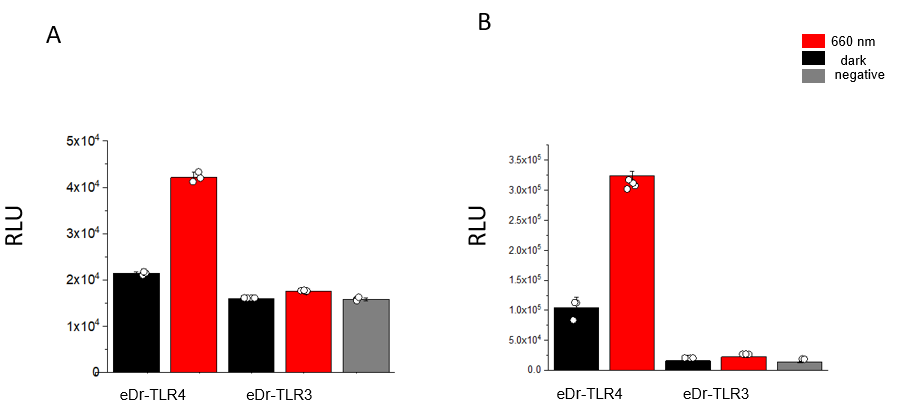
**

**Supplementary Figure 4.** Activation of the ISRE signaling in mammalian cells cotransfected with eDrTLRs and pEGFP-N1. **A)** Activation of the ISRE-dependent luciferase signaling with eDrTLR4 and eDrTLR3 in PC63 cells **B)** Activation of the ISRE-dependent firefly luciferase expression in HeLa cells. RLU – relative luminescence units. Data are presented as mean ± SD from (n=3) independent experiments. No statistical analysis was carried out.


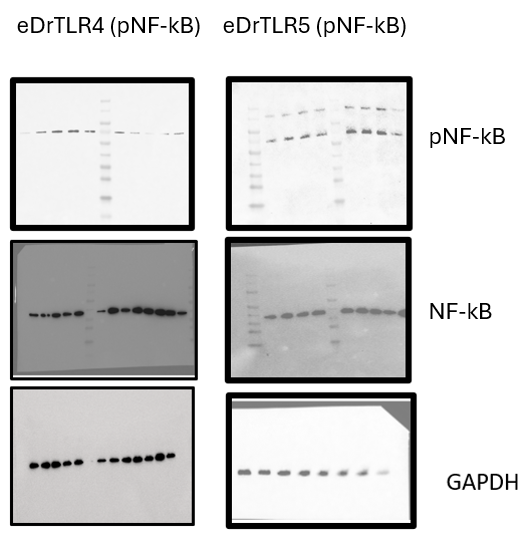


**Supplementary Figure 5. Uncut images of Western blots for Figure 4A and 4B.** See the main text for details.

**
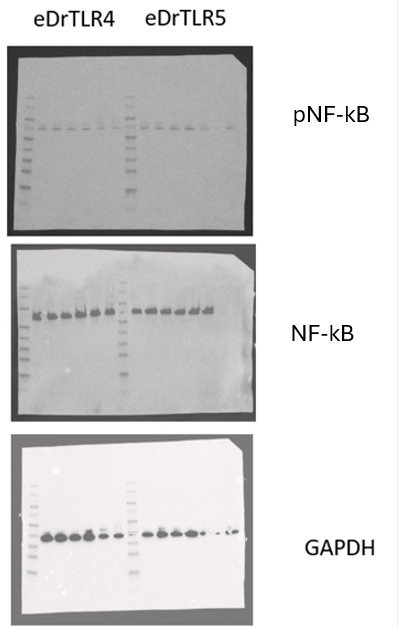
**

**Supplementary Figure 6. Uncut images of Western blots for Figure 4C.** See the main text for details.

**Supplementary Table 1. Ratios of RLU values before and after subtracting negative control.**

| **Construct used and miRNAs ratio** | **Fold of light induction** | **Fold of light induction (negative control is subtracted)** |
| --- | --- | --- |
| **NF-κB activation with miR-122/221 mediated SOCS inhibition in PC6-3 cells** | | |
| **eDr-2yf-her2tm**  5 ng miR122 +5ng miR221 | 2.7 | 4.06 |
| **eDr-2yf-her2tm**  25 ng miR122 +25ng miR221 | 3.04 | 5.18 |
| **eDr-2yf-her2tm**  100 ng miR122 +100 ng miR221 | 4.06 | 7.15 |
| **eDrher2TLR4**  25 ng miR122 +25ng miR221 | 2.15 | 4.74 |
| **eDrTLR4**  25 ng miR122 +25ng miR221 | 6.07 | 8.2 |
| **eDrTLR5**  25 ng miR122 +25ng miR221 | 1.8 | 5.7 |
| **NF-κB activation with miR-122/221 mediated SOCS inhibition in HeLa cells** | | |
| **eDrTLR4**  25 ng miR122 +25ng miR221 | 7.15 | 5.5 |
| **eDrTLR5**  25 ng miR122 +25ng miR221 | 3.7 | 11.5 |
| **ISRE (IRF3/7) activation with miR-122/221 mediated SOCS inhibition in PC6-3 cells** | | |
| **eDrTLR4**  25 ng miR122 +25ng miR221 | 1.9 | 4.7 |
| **eDrTLR3**  25 ng miR122 +25ng miR221 | 1.09 | 7.6 |
| **ISRE (IRF3/7) activation with miR-122/221 mediated SOCS inhibition in HeLa cells** | | |
| **eDrTLR4**  25 ng miR122 +25ng miR221 | 3.11 | 3.4 |
| **eDrTLR3**  25 ng miR122 +25ng miR221 | 1.4 | 3.8 |

**Supplementary Table 2. Full amino acid sequences of the eDrTLR constructs engineered in this study.**

| **eDrTLR3**  **METDTLLLWVLLLWVPGSTGDSKGEEDNMAIIKEFMRFKVHMEGSVNGHEFEIEGEGEGRPYEGTQTAKLKVTKGGPLPFAWDILSPQFMYGSKAYVKHPADIPDYLKLSFPEGFKWERVMNFEDGGVVTVTQDSSLQDGEFIYKVKLRGTNFPSDGPMQKKTMGWEASSERMYPEDGALKGEIKQRLKLKDGGHYDAEVKTTYKAKKPVQLPGAYNVNIKLDITSHNEDYTIVEQYERAEGRHSTGGMDELYKMSRDPLPFFPPLYLGGPEITTENCEREPIHIPGSIQPHGALLTADGHSGEVLQMSLNAATFLGQEPTVLRGQTLAALLPEQWPALQAALPPGCPDALQYRATLDWPAAGHLSLTVHRVGELLILEFEPTEAWDSTGPHALRNAMFALESAPNLRALAEVATQTVRELTGFDRVMLYKFAPDATGEVIAEARREGLHAFLGHRFPASDIPAQARALYTRHLLRLTADTRAAAVPLDPVLNPQTNAPTPLGGAVLRATSPMHMQYLRNMGVGSSLSVSVVVGGQLWGLIACHHQTPYVLPPDLRTTLEYLGRLLSLQVQVKEAADVAAFRQSLREHHARVALAAAHSLSPHDTLSDPALDLLGLMRAGGLILRFEGRWQTLGEVPPAPAVDALLAWLETQPGALVQTDALGQLWPAGADLAPSAAGLLAISVGEGWSECLVWLRPELRLEVAWGGATPDQAKDDLGPRHSFDTYLEEKRGYAEPWHPGEIEEAQDLRDTLTGALGELTVALILGIFLGTFIAFWVVYLL**EGWRISFYWNVSVHRVLGFKEIDRQTEQFEYAAYIIHAYKDKDWVWEHFSSMEKEDQSLKFCLEERDFEAGVFELEAIVNSIKRSRKIIFVITHHLLKDPLCKRFKVHHAVQQAIEQNLDSIILVFLEEIPDYKLNHALCLRRGMFKSHCILNWPVQKERIGAFRHKLQVALGSKNSVH |
| --- |
| **eDrTLR4**  **METDTLLLWVLLLWVPGSTGDSKGEEDNMAIIKEFMRFKVHMEGSVNGHEFEIEGEGEGRPYEGTQTAKLKVTKGGPLPFAWDILSPQFMYGSKAYVKHPADIPDYLKLSFPEGFKWERVMNFEDGGVVTVTQDSSLQDGEFIYKVKLRGTNFPSDGPMQKKTMGWEASSERMYPEDGALKGEIKQRLKLKDGGHYDAEVKTTYKAKKPVQLPGAYNVNIKLDITSHNEDYTIVEQYERAEGRHSTGGMDELYKMSRDPLPFFPPLYLGGPEITTENCEREPIHIPGSIQPHGALLTADGHSGEVLQMSLNAATFLGQEPTVLRGQTLAALLPEQWPALQAALPPGCPDALQYRATLDWPAAGHLSLTVHRVGELLILEFEPTEAWDSTGPHALRNAMFALESAPNLRALAEVATQTVRELTGFDRVMLYKFAPDATGEVIAEARREGLHAFLGHRFPASDIPAQARALYTRHLLRLTADTRAAAVPLDPVLNPQTNAPTPLGGAVLRATSPMHMQYLRNMGVGSSLSVSVVVGGQLWGLIACHHQTPYVLPPDLRTTLEYLGRLLSLQVQVKEAADVAAFRQSLREHHARVALAAAHSLSPHDTLSDPALDLLGLMRAGGLILRFEGRWQTLGEVPPAPAVDALLAWLETQPGALVQTDALGQLWPAGADLAPSAAGLLAISVGEGWSECLVWLRPELRLEVAWGGATPDQAKDDLGPRHSFDTYLEEKRGYAEPWHPGEIEEAQDLRDTLTGALGELTVALILGIFLGTFIAFWVVYLL**KFYFHLMLLAGCIKYGRGENIYDAFVIYSSQDEDWVRNELVKNLEEGVPPFQLCLHYRDFIPGVAIAANIIHEGFHKSRKVIVVVSQHFIQSRWCIFEYEIAQTWQFLSSRAGIIFIVLQKVEKTLLRQQVELYRLLSRNTYLEWEDSVLGRHIFWRRLRKALLDGKSWNPEGTVGTGCNWQEATSI |
| **eDrTLR5**  **METDTLLLWVLLLWVPGSTGDSKGEEDNMAIIKEFMRFKVHMEGSVNGHEFEIEGEGEGRPYEGTQTAKLKVTKGGPLPFAWDILSPQFMYGSKAYVKHPADIPDYLKLSFPEGFKWERVMNFEDGGVVTVTQDSSLQDGEFIYKVKLRGTNFPSDGPMQKKTMGWEASSERMYPEDGALKGEIKQRLKLKDGGHYDAEVKTTYKAKKPVQLPGAYNVNIKLDITSHNEDYTIVEQYERAEGRHSTGGMDELYKMSRDPLPFFPPLYLGGPEITTENCEREPIHIPGSIQPHGALLTADGHSGEVLQMSLNAATFLGQEPTVLRGQTLAALLPEQWPALQAALPPGCPDALQYRATLDWPAAGHLSLTVHRVGELLILEFEPTEAWDSTGPHALRNAMFALESAPNLRALAEVATQTVRELTGFDRVMLYKFAPDATGEVIAEARREGLHAFLGHRFPASDIPAQARALYTRHLLRLTADTRAAAVPLDPVLNPQTNAPTPLGGAVLRATSPMHMQYLRNMGVGSSLSVSVVVGGQLWGLIACHHQTPYVLPPDLRTTLEYLGRLLSLQVQVKEAADVAAFRQSLREHHARVALAAAHSLSPHDTLSDPALDLLGLMRAGGLILRFEGRWQTLGEVPPAPAVDALLAWLETQPGALVQTDALGQLWPAGADLAPSAAGLLAISVGEGWSECLVWLRPELRLEVAWGGATPDQAKDDLGPRHSFDTYLEEKRGYAEPWHPGEIEEAQDLRDTLTGALGELTVALILGIFLGTFIAFWVVYLL**TKFRGFCFICYKTAQRLVFKDHPQGTEPDMYKYDAYLCFSSKDFTWVQNALLKHLDTQYSDQNRFNLCFEERDFVPGENRIANIQDAIWNSRKIVCLVSRHFLRDGWCLEAFSYAQGRCLSDLNSALIMVVVGSLSQYQLMKHQSIRGFVQKQQYLRWPEDLQDVGWFLHKLSQQILKKEKEKKKDNNIPLQTVATIS |

Red - mCherry with the N-terminal Igκ signal. Blue – DrBphP-PCM. Green – artificial transmembrane domain. Black – signaling intracellular TIR domain.
